# An Anatomical and Experimental Study of Sacral Anesthesia

**Published:** 1918-04

**Authors:** 


***An Anatomical and Experimental Study of Sacral Anesthesia.***

By James E. Thompson, of *Galveston*. Abs. from the *Annals of Surgery*, Dec. 1917.

The author considers sacral anesthesia the best form of local anesthesia. He injects 30 cc. of the solution[1] into the sacral canal and gives it time to diffuse in the space outside the dura mater, where it bathes peripheral nerves before they pass out through the intervertebral foramina.

The region of the anus is first affected. The result is more rapid if the nerve trunk lies near to the bulk of the fluid injected. The region supplied by the sacral nerves from the second downward is chiefly affected; but the anesthesia often extends upward beyond the second sacral. In women the clitoris and labia minora do not often lose their sensibility completely.

The sacral needle seldom passes higher than the third sacral vertebra, and so the bulk of the fluid is injected at this level, producing its maximum effect in the sacral nerves. Sometimes sensation and motion are lost in both legs and anesthesia is complete as high as the lower thoracic nerves.

From injections of stains upon cadavers the following observations were made:

In only one case was there a failure to inject, caused by a curved sacrum and an unusually narrow and flattened canal.

In two cases in which the dura was exposed at the level of the seventh cervical vertebra, the outer surface of the dura was so deeply stained as to suggest staining at a much higher level.

In no body was any of the injection fluid found inside the dura mater.

In every case there was deep staining of both the anterior and posterior branches of the sacral nerves.

In not a single case did the coloring matter gain entrance to the subarachnoid space.

1. Three No. A tablets are dissolved in 30 cc. of distilled water and 10 drops of a 50 o/o solution of calcium chloride are added. The composition of the No. A tablets is novocaine, 0.125 gm.; suprarenine 0.000125 gm. One injection is usually sufficient.

In another series of experiments, a needle 6.3 cm. long was employed. 30 cc. of an aqueous solution was injected into the sacral canals. In one case, in which the injection was extra-dural, the stain had passed as high as the attachment of the dura mater to the margin of the foramen magnum. In two cases it had extended as high as the cervical segment of the spine.

In one case in which the stain had not passed higher than the lower end of the dural sheath opposite the second sacral vertebra, the stain had passed into the vertebral veins, which were found to be particularly large and dilated. In other cases the stain extended on an average to the level of the ninth thoracic segments. As a rule, very little difficulty is met with clinically in passing the needle into the sacral canal. In fat people the bony landmarks are obscure and in cases in which the coccyx is ankylosed with the sacrum, an extra allowance must be made. The direction of the needle should be upward, exactly in the middle line, towards a point about 2 cm. deeper than the prominent process of the first sacral vertebra. It is not necessary to introduce the needle farther than 3 or 4 cm., although our experiments prove that the risk of wounding the dura is very slight.

The operation may be rendered painless by the infiltration of a 25 o/o solution of novocaine into the skin and subcutaneous tissues over the hiatus.

In 24 cases out of 33 in which the sacra were examined, the probe passed along the whole canal. In 8 cases it passed at least as high as the second sacral foramen. In 1 case it passed along the canal for a distance of 2.5 cm. and reached as high as the fourth sacral foramen.

